# The balance between proinflammatory, “bad”, and immunomodulatory, “good”, lipopolysaccharide for understanding gut-derived systemic inflammation

**DOI:** 10.3389/fimmu.2025.1588129

**Published:** 2025-07-09

**Authors:** Rafael Tume, Samar El Sherbiny, Roberto Bono, Thomas Gautier, Jean Paul Pais de Barros, Tomás Meroño

**Affiliations:** ^1^ Biomarkers and Nutrimetabolomics Laboratory, Department de Nutrició, Ciències de l’Alimentació i Gastronomia, Nutrition and Food Safety Research Institute (INSA), Facultat de Farmàcia i Ciències de l’Alimentació, Universitat de Barcelona, Barcelona, Spain; ^2^ Department of Public Health and Pediatrics, University of Turin, Turin, Italy; ^3^ Center for Translational and Molecular Medicine (CTM) UMR1231, Inserm/Université Bourgogne Europe, Lipness Team, Dijon, France; ^4^ DiviOmics Platform, UMS 58 BioSand – Université Bourgogne Europe, Dijon, France; ^5^ Centro de Investigación Biomédica en Red de Fragilidad y Envejecimiento Saludable (CIBERFES), Instituto de Salud Carlos III, Madrid, Spain

**Keywords:** lipopolysaccharide (endotoxin), inflammation, intestinal permeability, nutrition, gut microbiota

## Abstract

Lipopolysaccharide (LPS) from Gram-negative bacteria has been one of the most studied pathogen-associated molecular patterns triggering rapid inflammatory reactions. However, evidence shows that not all LPS molecules are proinflammatory (“bad”), and that “good” LPS from gut commensal bacteria exert immunomodulatory actions. The Limulus amebocyte lysis test commonly used to quantify LPS in circulation, only targets “bad” LPS, when not inactivated by plasma components. Use of other methods showed healthy subjects featuring elevated levels of LPS (suggesting predominance of “good” or inactive LPS in circulation). This review aims to summarize the evidence supporting the higher abundance of “good” LPS coming from gut commensals of healthy individuals and their potential impact in human health.

## Introduction

### Lipopolysaccharides: structure, function, and heterogeneity

Lipopolysaccharides (LPS) are amphipathic glycoconjugates that serve as an essential component of the outer membrane of Gram-negative bacteria. In some cases, these bacteria can comprise up to approximately 65% of the intestinal microbiota, with the majority of LPS being derived from the Bacteroidota and Pseudomonadota (formerly *Proteobacteria*) phyla (1). LPS performs essential structural functions by providing integrity to the outer membrane and protecting the bacterial cell from adverse conditions (2). Additionally, LPS facilitates adhesion to surfaces through biofilm formation, increasing resistance to antibiotics and promoting colonization. One of its most notable characteristics is its thermal stability, as well as its key role in the pathogenicity of Gram-negative bacteria, distinguishing it from bacterial exotoxins. This is why LPS is also referred to as “endotoxin”. LPS is constantly released during bacterial division and cell lysis, making it a ubiquitous toxin in the environment.

#### Structure of LPS and LPS types

**Figure 1:**
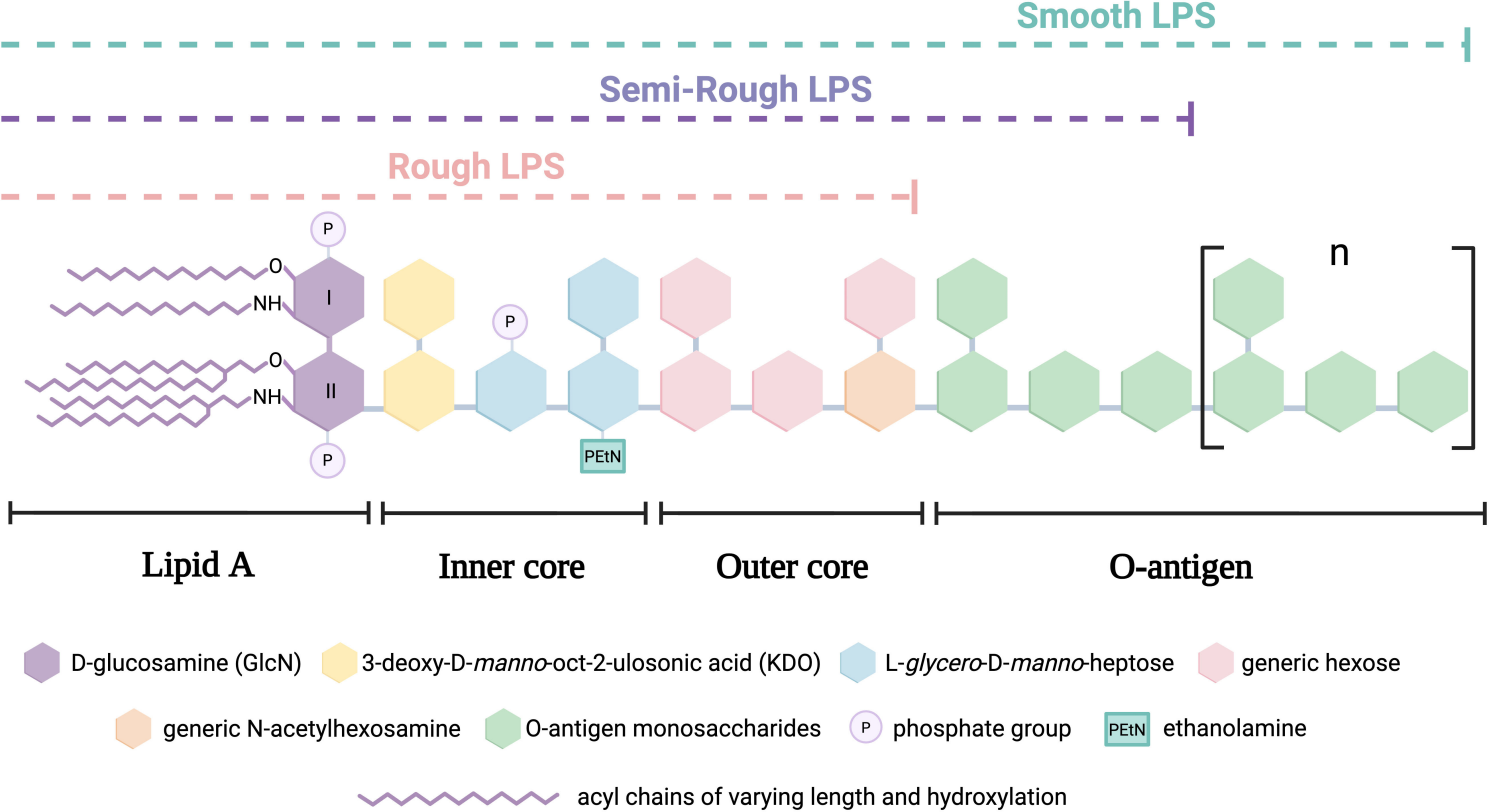
Schematic structure of canonical lipopolysaccharide (LPS) types in Gram-negative bacteria.

LPS exhibit structural variations among different bacteria and even among strains of the same species. However, in general terms, they are composed of the following main regions: (**Figure 1**):

- *Lipid A*: The hydrophobic portion anchored to the membrane, which serves as the primary virulence factor by binding to Toll-like receptor 4 (TLR4), triggering an inflammatory signaling cascade. It contains, among others, several acyl chains, including specific hydroxylated fatty acids allowing the branching of secondary acyl chains via ester bonds.
- *Core oligosaccharide*: An intermediate hydrophilic segment consisting of an inner and outer core, which connects lipid A to the O-antigen.
- *O-antigen*: A highly variable distal polysaccharide composed of repetitive units of up to eight distinct sugars. It is the main determinant of bacterial antigenicity and serotyping (e.g., *Escherichia coli* presents around 170 serotypes) (3).

Additionally, LPS can be classified mainly into two types based on the presence and structure of the O-antigen:

- Smooth LPS (S-LPS): Contains the O-antigen, making it less susceptible to binding with plasma lipoproteins, favoring its circulation and prolonging its inflammatory activity.
- Rough LPS (R-LPS): Lacks the O-antigen and consists only of lipid A and the core oligosaccharide. It has a higher affinity for plasma lipoproteins, facilitating its neutralization and reducing its inflammatory effect. A related intermediate form, called semi-rough LPS, contains a single O-antigen repeat attached to the core and last, a specific subtype of R-LPS is lipooligosaccharide (LOS), characterized by the lack of O-antigen and a compact, structurally defined core oligosaccharide that serves as the main antigenic determinant (4).

#### Main determinants of LPS heterogeneity

To date, the structures of LPS from more than 50 bacterial species have been well characterized, revealing significant diversity in their structural modifications (5). Among these, LPS heterogeneity is determined by multiple structural factors, the most relevant being:

- Number and type of acyl chains in lipid A: The length, branching and degree of saturation of these chains influence immune system activation. Some variants can even modulate or inhibit immune activation (6–9).
- O-antigen length: Among structures sharing the same type of lipid A, a longer polysaccharide chain promotes the formation of stable aggregates in plasma, prolonging circulation time and enhancing inflammatory potential (10).
- Modifications with functional groups: Phosphate and ethanolamine groups can bind to Lipid A or the core oligosaccharide, altering interactions with immune cells and receptors (11).

### Intestinal exposure and LPS translocation into the circulation

Under normal physiological conditions, very low levels of LPS can be found in plasma (12, 13). This is because a healthy intestine restricts the passage of LPS molecules into the bloodstream, despite an estimated 1 g of LPS being present in the intestinal lumen (14). In spite of this constant exposure to LPS, enterocytes and colonocytes exhibit a hyporesponsive state to these stimuli, partly explained by the low expression of Toll-like receptor 4 (TLR4) in these cells (15, 16). This intestinal tolerance mechanism minimizes inflammatory responses in the intestinal lumen (14). Additionally, exposure to LPS from gut microbiota plays a crucial role in immune system development and maturation, training regulatory T cells within the Gut-Associated Lymphoid Tissue (GALT) (17). An individual’s LPS profile depends on gut microbiota composition, which varies according to geographic and lifestyle factors, among others (18, 19).

Given this, LPS can enter systemic circulation through three main mechanisms:

- Infection by exogenous Gram-negative bacteria.
- Paracellular pathway, facilitated by increased intestinal permeability.
- Transcellular transport, where LPS is absorbed by enterocytes after dietary fat intake and secreted in association with chylomicrons at the basolateral membrane (13, 20, 21). Through the lymphatic system, chylomicron-borne LPS eventually reaches the bloodstream.

In relation to transcellular transport, it has been reported that after a single high-fat meal, plasma LPS levels increase, peaking 1–5 hours post-ingestion (12, 22, 23). Interestingly, despite high-fat diets being associated with an increase in Bacillota (formerly Firmicutes, Gram-positive bacteria) and a reduction in Bacteroidota (Gram-negative bacteria), elevated circulating LPS levels have been observed in humans under high-fat diets (24, 25) or other unhealthy dietary patterns (26, 27). These individuals can exhibit LPS levels 2 to 3 times higher compared to those on healthy diets. This phenomenon is referred to as “metabolic endotoxemia”, as LPS levels significantly rise without reaching the extreme levels observed in sepsis and it may have a prominent role in the development of cardiometabolic diseases (20, 28). In addition to diet, obesity also influences the magnitude of metabolic endotoxemia, with more pronounced increases in chylomicron-associated LPS levels as well as inflammatory cytokine production after a high fat meal in subjects with obesity (29). Another possible mechanism linking high-fat diets and LPS burden is related to altered intestinal permeability due to alteration in gut microbiome composition. By reducing *Bifidobacterium* levels, high-fat diets decrease glucagon-like peptide-2 (GLP-2) production, impairing intestinal barrier function and increasing paracellular LPS translocation (30, 31). This consequent endotoxemia can then promote the production of proinflammatory cytokines such as TNF-α, IL-6, and IL-1β, further increasing intestinal permeability. This vicious cycle may contribute to metabolic dysfunction and low-grade chronic systemic inflammation (32, 33). Nonetheless, the relative contributions of transcellular and paracellular pathways for the total metabolic endotoxemia burden remain to be determined.

### LPS metabolism: transport, biological activity, and elimination

Once in circulation, LPS can follow two main metabolic pathways:

- Immune activation: Binding to LPS-binding protein (LBP), which transfers LPS to the CD14/MD-2/TLR4 complex, triggering inflammatory signaling.
- Neutralization and elimination: Transfer of LPS to lipoproteins, mediated by LBP and phospholipid transfer protein (PLTP), followed by hepatic metabolism and biliary excretion.

#### Immune system activation via the CD14/MD-2/TLR4 complex

In an extracellular recognition mechanism, LPS initially binds to LBP. Then, LBP transfers LPS to the CD14 receptor, which exists in both soluble (sCD14) and membrane-bound forms (34). Since TLR4 cannot directly recognize LPS, CD14 facilitates its transfer to myeloid differentiation factor-2 (MD-2), stabilizing LPS binding and allowing TLR4 dimerization, which activates the signaling cascade (35). This CD14/MD-2/TLR4 complex is mainly present in immune cells such as monocytes, macrophages, and dendritic cells, though it is also detected in B cells, hepatocytes, adipocytes, and muscle cells, where it plays a role in inflammatory and metabolic responses.

The activation of this complex triggers two main signalling pathways:

1. The MyD88 pathway (rapid proinflammatory response): Activates myeloid differentiation primary response protein 88 (MyD88), leading to the activation of the NF-κB pathway and of the NLRP3 inflammasome (36), and eventually to the production of proinflammatory cytokines such as TNF-α, IL-6, and IL-1β (37).
2. The TRIF pathway (sustained antiviral response): Through the TIR-domain-containing adapter-inducing interferon-β (TRIF) adaptor protein, this cascade induces the production of IFN-β and other interferon-stimulated genes (ISGs), promoting an antiviral response and modulating inflammation in a more sustained manner (38, 39).

#### Intracellular immune recognition of LPS

In addition, recent evidence highlights an alternative intracellular recognition pathway for LPS. Certain immune cells, including macrophages, can internalize LPS through multiple mechanisms, such as endocytosis of outer membrane vesicles (OMVs) released by Gram-negative bacteria or direct uptake of LPS-protein complexes via endosomal pathways. Once inside the cytoplasm, LPS is recognized by inflammatory caspases, which bind directly to cytosolic LPS, triggering once again the activation of the NLRP3 inflammasome as well as the maturation of gasdermin D. This leads to the maturation and secretion of IL-1β and IL-18, as well as the gasdermin D-induced pyroptotic cell death (40), which serves as an additional immune defense mechanism. However, in conditions like sepsis, excessive activation of this pathway can contribute to systemic inflammation and tissue damage (41, 42).

#### Neutralization and elimination of LPS via lipoproteins

Since LPS are amphipathic phospholipids, in addition to binding to LBP, they can also bind to PLTP, facilitating their transfer to plasma lipoproteins, particularly HDL (43–45). This process limits LPS binding to TLR4, thereby exerting a protective effect against endotoxin-mediated inflammation (46, 47). It has also been reported that after 40 minutes of exposure, more than 50% of circulating LPS has been neutralized via this pathway, and that even after 10 minutes, LBP preferentially presents LPS to HDL rather than to CD14 in immune cells (48).

Once bound to HDL, LPS is transported to the liver, where it can be taken up mostly by hepatocytes or Kupffer cells. LPS uptake by hepatic cells was shown to involve scavenger receptor class-B type1 (SR-BI) which is the canonical receptor for HDL (49). In Kupffer cells, LPS undergoes processing through deacylation via acyl-oxyacyl hydrolase (AOAH). This enzyme is also active in other immune cells, such as macrophages and dendritic cells, which are present in various tissues, including the intestinal *lamina propria*. Finally, the resulting LPS degradation products, as well as remaining whole LPS, are excreted in the feces via bile, although this process can take several days (50).

Although HDL may be the main lipoprotein class involved in LPS detoxification, triglyceride-rich lipoproteins (QM and VLDL), and LDL also play a significant role in this process. Indeed, all lipoprotein classes are able to bind LPS (44) and to alleviate the inflammatory response of LPS-treated macrophages *in vitro* (51, 52) and to protect against the noxious effects of LPS in animal models (53). In addition to the role of SR-BI in the clearance of HDL-bound LPS, the involvement of the LDL receptor (LDLR) in LPS detoxification and protection against infection has been demonstrated in LDLR-deficient mice (54) and in humans harboring mutations of PCSK9, the endogenous inhibitor of LDLR (55). Overall, these data show that the different lipoprotein classes offer complementary clearance pathways for circulating LPS, with possible interconnections between them.

### LPS structural variations and their impact on immune activation

As previously described, the classical activation of the immune system by LPS is mediated through its interaction with the TLR4/MD-2 complex, with distinct immunological outcomes depending on structural variations in LPS. In the case of the degree of acylation of lipid A, a hexa-acylated lipid A, like the one from *E. coli* LPS, typically contains four primary and two secondary acyl chains and induce a strong inflammatory response. This is because the first five acyl chains are inserted deeply into a hydrophobic pocket within MD-2, while the sixth chain extends outward and facilitates the recruitment of a second TLR4 monomer. This interaction is essential for the dimerization of the TLR4/MD-2 complex, a prerequisite for downstream signaling activation (56). In contrast, tetra- and penta-acylated lipid A (such as those from gut commensals belonging to the *Bacteroides* genus) exhibit markedly reduced immune activation due to their limited ability to promote TLR4 dimerization, as the missing acyl chains impair the necessary structural interactions (1, 57, 58). However, not only the number but also the type of acyl chains (length and degree of saturation) influences bacterial activation of the immune system (35). For example, it has been observed that variants of lipid A from *E. Coli* with acyl chains of 12 to 14 carbons are more endotoxic than variants with longer chains of 16 to 18 carbons by an impairment of LPS interaction with the CD14/MD-2/TLR4 complex (59–61).

Additionally, the number of phosphate groups also plays an important role, as they increase the negative charge of LPS, facilitating its binding to proteins required for LPS presentation to TLR4 and also to TLR4 itself. Thus, hexa-acylated lipid A (like *E. coli* one) generally contains two negatively charged phosphate groups, which interact via ionic bonds with positively charged amino acid residues in the TLR4/MD-2 complex, thereby stabilizing it (62, 63). In contrast, LPS derived from *Bacteroides vulgatus* (a common gut commensal), in addition to being hypoacylated and containing long-chain fatty acids, is deficient in these critical phosphate modifications, which further reduces its ability to trigger a strong TLR4-mediated immune response, underlining the importance of this aspect for immune modulation (63). The differential signaling of lipid A depending on its degree of phosphorylation has been exploited, for example, in the design of monophosphoryl lipid A (MPLA). This is a semi-synthetic derivative of LPS from Salmonella, in which one of the two phosphate groups has been removed. As a result, it acts as a partial TLR4 agonist, primarily triggering MyD88-dependent signaling with minimal TRIF activation, thereby leading to reduced inflammation. Because of its safety and efficacy profile, MPLA has been approved by the FDA as an adjuvant in vaccines such as those against HPV and herpes zoster (64, 65). Also, gut cells present an intestinal isoenzyme of alkaline phosphatase (iALP) at the brush border which participates in the hydrolysis of the phosphate groups of the LPS molecule. Indeed, in animal models, overexpression of iALP has been associated with protection from atherosclerosis through inactivation of LPS inflammatory actions and preservation of the intestinal barrier function (66).

Many of the structural modifications discussed above result in what is known as weak agonistic LPS. Unlike strong agonists or pure antagonists, weak agonistic LPS induce low-level receptor signaling, which can promote anti-inflammatory effects. It is important to notice that similar LPS modifications are part of the immune evasion strategies exploited by certain pathogens such as *Yersinia pestis* (67), *Francisella novicida* (68), *Porphyromonas gingivalis* and *Helicobacter Pylori* (69).

A summary of the structural characteristics of LPS from different gut commensals is summarized in **Table 1**.

**Table 1:** Comparative Overview of LPS Structural features and immunogenic potential of Gut Commensals.

Gut commensal	Typical Lipid A chain length	Number of acyl-chains	Phosphate substituents	Type	Inmunogenic potential	Reference
*Akkermansia* *muciniphila*	14,15,17	4-6	1-2	LOS	Immunomodulatory	(70)
*Bacteorides* *Dorei*	15-17	5	1	NR	Immunomodulatory	(18, 71)
*Bacteroides eggerthii*	15-17	4-5	1-2	LOS	Immunomodulatory	(72)
*Bacteroides* *fragilis*	15-17	5	1	S-LPS	Immunomodulatory	(71, 73)
*Bacteroides* *ovatus*	NR	5	1	NR	Immunomodulatory	(71)
*Bacteroides thetaiotaomicron*	15-17	5	1	LOS	Immunomodulatory	(71, 73, 74)
*Bacteroides vulgatus*	15-17	4-5	1	S-LPS	Immunomodulatory	(63, 71)
*Escherichia Coli*	12,14	6	2	S-LPS	Proinflammatory	(75)
*Parabacteroides* *distasonis*	NR	NR	NR	NR	Immunomodulatory	(76)
*Parabacteroides goldsteinii*	NR	5	1	NR	Immunomodulatory	(77)
*Desulfovibrio desulfuricans*	12,14 (16)	5-6	2	S-LPS	Null to proinflammatory	(78, 79)
*Desulfovibrio vulgaris*	15-17	5	NR	NR	NR	(80, 81)
*Veillonella parvula*	13	6	1-2	S-LPS*	Immunomodulatory	(82)

NR, Not reported; S-LPS, smooth-lipopolysaccharide; R-LPS, rough-lipopolysaccharide; LOS, lipooligosaccharide.

*R-LPS may be present in other non-intestinal strains.

Although previous authors have employed different names to refer to immunomodulatory LPS (such as physiologic LPS, or anti-inflammatory LPS, etc.) (83–85), we consider for reaching a wider audience and to introduce the concept in this upbreak of gut microbiome research to classify them as “good” LPS as opposed to the largely known proinflammatory, “bad” LPS.

#### “Bad” vs. “Good” LPS: Gut commensals LPS and potential impact for human health

Evidence from metagenomic data of the Human Microbiome Project have confirmed that immune silencing via LPS is an intrinsic feature of the human gut microbiome (1). In healthy subjects, the majority of LPS primarily originates from bacteria of the phylum Bacteroidota (including genera such as *Bacteroides, Parabacteroides*, *and Prevotella*) rather than from Pseudomonadota, which includes *Escherichia, Shigella, Enterobacter*, among others (1, 86). Therefore, in opposition to what is commonly known about LPS, gut commensals LPS is not universally harmful, especially considering that most Gram-negative commensal bacteria are non-pathogenic and produce LPS, as well.

The mechanism that most probably drives these beneficial effects of LPS can be competition for TLR4 receptors. This seems to be exerted in two main ways: by directly competing with proinflammatory LPS in the binding with TLR4 or by preventing this binding by inhibiting their interaction (83). Additionally, some LPS are immunosilent, meaning they do not elicit any immune response, either pro or anti-inflammatory (1, 18).

LPS from *Bacteroides* members (likely the most abundant in the gut environment) are characterized by a hypoacylated lipid A structure (mainly penta-acylated), probably due to the absence of the late acyltransferase LpxM in the Raetz pathway, which normally adds the sixth acyl chain (71). These LPS are also monophosphorylated and contain 15–17-carbon acyl chains (5, 72, 74), all of which generally confer immunomodulatory effects. For example, administration of LPS from *B. vulgatus* demonstrated a direct anti-inflammatory effect by stimulating IL-10 production (63), while also reducing colon inflammation by inducing endotoxin tolerance (7). In combination with LPS from *B. dorei*, it may also confer protection against atherosclerosis (87). Similarly, LPS from *B. fragilis* and *B. ovatus* have been shown to improve intestinal inflammation and permeability in mice (88). In addition, Grombach et al. reported in a murine model that a microbiota dominated by *Bacteroides*, as opposed to one dominated by *Enterobacteriaceae*, induced lower levels of inflammation and immune activation in chemically induced colitis (61).

Regarding LPS from Parabacteroides, oral administration of a membrane fraction from *P. distasonis, expected to contain LPS*, not only exerted anti-inflammatory effects (89) but purified LPS from this species also reduced chemically induced acute colitis by inhibiting TNF-α production (76). Likewise, LPS from *P. goldsteinii* demonstrated an anti-inflammatory effect, reducing the development of induced chronic obstructive pulmonary disease (COPD) (77) and ameliorating *H. pylori*-related inflammation (90) presumably due to its hypoacylated lipid A structure (91). As for *Prevotella*, although approximately 40 species have been described, most belong to the oral microbiota, with *Prevotella copri* being the most prominent species in the gut. While its LPS has not been extensively characterized to date, current evidence suggests it may act as a pro-inflammatory molecule, as it is associated with increased LPS biosynthesis in the gut and has been linked to vascular calcification and metabolic liver disease (92, 93).

Although most bacteria within the Bacillota phylum are Gram-positive, there are exceptions such as *Veillonella*, a Gram-negative bacterium that therefore contains LPS. Oral administration of this bacterium provided protection against asthma in a mouse model of induced asthma. Moreover, higher levels of *Veillonella* have been reported in healthy children compared to those with asthma, with its LPS being identified as the main contributing factor (94).

Another interesting case is *Akkermansia muciniphila*, a Gram-negative bacterium to which several beneficial effects on the intestinal barrier have been attributed. This bacterium was recently approved by the EFSA as a novel food (95), specifically in its pasteurized form, highlighting that its beneficial effects may lie in its structural components rather than in its metabolic activity. In this context, its hypoacylated and monophosphorylated LOS, which lacks an O-antigen, favors an immunomodulatory response (70).

These findings indicate that weak agonistic LPS may serve as a potential therapeutic strategy providing an alternative to conventional treatments. Summary of the immunogenic potential of LPS from gut commensals characterized so far can be found at **Table 1**. These findings underscore not only the critical role of gut microbiota composition but also the therapeutic potential of microbiome-derived LPS in promoting immune homeostasis and mitigating inflammation in age-related diseases. In this regard, Gaber et al. (96) showed that in postmenopausal women gut microbiome alterations (with an imbalance favoring proinflammatory LPS producing bacteria, *Escherichia coli*, *Shigella* spp., and *Klebsiella* spp.) may contribute to visceral adiposity and that LPS from mice following a low-fat diet prevented some of the cardiometabolic alterations induced by a high-fat diet, underscoring the potential of “good” LPS for treatment of cardiometabolic diseases.

### Approaches to LPS measurement in humans

From the above-mentioned, it arises the need for methods to characterize circulating LPS molecules at higher detail, reflecting the balance between “good” and “bad” LPS. LPS measurement is methodologically challenging, and several studies have shown the major limitation of the commonly used Limulus Amebocyte Lysate (LAL) test. LPS levels measured through LAL assays decrease in time because these assays measure the bioactive, free LPS molecules, which are rapidly detoxified by LBP, PLTP and lipoproteins in serum (97). Consequently, alternative biomarkers such as the levels of LBP, titers of antibodies against LPS (endoCab) or soluble CD-14 have been used. Nonetheless, these provide indirect evidence of higher exposure to LPS but neither contribute to characterize qualitative LPS alterations, nor to evaluate the balance between “good” and “bad” LPS.

Fatty acids in lipid A are 3-hydroxy fatty acids (3OH-FAs) of different chain lengths with a diverse acylation pattern. As already mentioned, the chain length of the acyl moieties composing the lipid A may be of relevance to explain differences among LPS immunogenic potential. For this reason, the measurement of esterified 3OH-FAs in circulation may be a proxy for LPS concentration, as these can only come LPS-Lipid A. GC/MS methods were initially used for the separation and quantification of these esterified 3OH-FAs (98). More recently, Pais-de-Barros et al. developed and optimized an HPLC/MS/MS methodology to detect and quantify esterified 3OH-FAs, obtained by calculating the difference between total and non-esterified (free) 3-OH-FAs, resulting in a LPS quantitative assay with increased sensitivity and accuracy (97). A recent study using this methodology showed that healthy controls exhibit higher concentrations of total esterified 3-OH-FAs than patients with end-stage renal disease (59). However, they identified that patients with end-stage renal disease exhibited a higher proportion of C12 and C14 3OH-FAs, which have been shown to produce a higher inflammatory activation in peripheral blood monocyte cells. Furthermore, studies done with this methodology show that among healthy subjects, the most prevalent esterified 3-hydroxy fatty acids are the C16 and C18 fatty acids. Therefore, this gives major evidence underscoring the importance of “good” LPS in human health. Alterations in LPS may therefore not only be quantitative, but most likely qualitative as well. These qualitative alterations may be relevant for disease development and progression and hold therapeutic potential.

## Discussion-future insights

In addition to the possibility of designing LPS for use in postbiotics, probiotics, or vaccine adjuvants (60, 65), potential applications of LPS derived from non-commensal bacteria such as *Rhodobacter capsulatus* and *Rhodobacter* sp*haeroides* (99) which exhibit TLR4-antagonistic activity, as well as *Pantoea agglomerans* (100), whose LPS acts as a weak TLR4 agonist, are also being explored. Moreover, besides a potential immunoregulatory role of early-life exposure to specific LPS (18, 94), the balance between “good” and “bad” commensal LPS may shape the host environment for the development of systemic low-grade inflammation. The probable factors affecting this balance may require the concerted impairment of both gut microbiota composition, and intestinal barrier. The role of “good” LPS exerting immunomodulatory actions might have been disregarded in the bibliography as methods for demonstrating its presence in circulation have been recently developed and are not widely distributed. As increased intestinal permeability has been linked to several age-related chronic diseases, LPS translocation may, in this particular context, be detrimental. However, approaches to increase the pool of “good” LPS could be of particular use against age-related diseases. An association between “healthy” plant-based diets and lower “bad” LPS concentrations have been reported (26, 101). Last, next-generation probiotics based on species of the *Bacteroides* genus, showing immunomodulatory LPS, have received safety clearance by the European Commission and authorized for food processing and are under study (102). In terms of Public Health, which is increasingly targeting chronic inflammation for prevention of chronic diseases, the present review highlights how diet and gut microbiome eubiosis are of major relevance for promoting health.
